# Effects of climate on pine processionary moth fecundity and on its egg parasitoids

**DOI:** 10.1002/ece3.1664

**Published:** 2015-10-31

**Authors:** Riziero Tiberi, Matteo Bracalini, Francesco Croci, Guido Tellini Florenzano, Tiziana Panzavolta

**Affiliations:** ^1^ Department of Agrifood Production and Environmental Sciences University of Florence Via Maragliano 77 – 50144 Florence Italy

**Keywords:** Encyrtidae, Eulophidae, Hymenoptera, Lepidoptera, Notodontidae, temperature

## Abstract

Climate change may be affecting the fecundity of phytophagous insects as well as impacting their natural enemies. However, temperature impacts these two insect groups differently, disrupting population regulation mechanisms, and ultimately, possibly culminating in an outbreak of the host. The pine processionary moth (PPM) is one of the most harmful insects of the Mediterranean basin. Not only are PPM larvae harmful to plants, but they are also dangerous to humans because of their urticating hairs. Although some information is available on climate change effects on the PPM, little is known about its potential effects on PPM egg parasitoids, especially on their distribution range or on their role in controlling PPM populations. The aim of this article was to verify the effects of climate on PPM fecundity and on its egg parasitoids. Our results show that climate warming may affect the PPM positively, but not its egg parasitoids. Specifically, during our study mild winters directly favored the PPM, while increasing summer temperatures (over 30°C) also favored the PPM indirectly, by decreasing parasitism rates. We predict that ever‐milder winters will not only favor PPM development, but also encourage it to spread in otherwise previously inhospitable environments.

## Introduction

Climate change affects phytophagous insects, as demonstrated by several scientific papers on the main forest insect species (Battisti 2008). In fact, higher winter temperatures influence not only these insects' survival, as is the case of the pine processionary moth (PPM), *Thaumetopoea pityocampa* (Denis et Schiffermüller) (Lepidoptera, Notodontidae) (Battisti et al. 2005), but also their fecundity (Berger et al. 2008). Furthermore, the synchrony between insects and host plants is disrupted (Hance et al. 2007). In addition, while most of these studies focused on climate change effects at the second trophic level, few studies of the third trophic level are available.

Climate change affects insect outbreaks not only directly, but also indirectly, through impacting their natural enemies (Stireman et al. 2005; Hance et al. 2007; Berggren et al. 2009; Klapwijk et al. 2012; Ma et al. 2014). Although effects of climate change on parasitoids and on their role in population regulation mechanisms are little known, parasitoids are presumably particularly susceptible to environmental changes, as they also depend on their hosts' resilience (Hance et al. 2007). Moreover, temperature flux affects phytophagous insects and their natural enemies differently, according to each one's temperature preferences (Hance et al. 2007). This disrupts population regulation mechanisms (Zovi et al. 2006).

**Figure 1:**
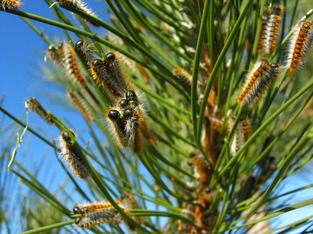
*Thaumetopoea pityocampa* larvae.

The PPM is a major pest in the Mediterranean basin due to their larvae (Fig. 1), which voraciously feed on pine needles and have urticating hairs. PPM larvae overwinter inside tents on host trees, which they periodically leave for feeding during autumn and spring, as well as throughout the winter if climatic conditions allow it (Huchon and Démolin 1970), stripping trees of leaves. In contrast, adults have a very short life span; during summer, a few hours after emerging, females mate and lay a single egg mass, then they die (Huchon and Démolin 1970; Pérez‐Contreras and Soler 2004). Not only are PPM's defoliating larvae harmful to plants (Devkota and Schmidt 1990; Kanat et al. 2005; Arnaldo et al. 2010), but their urticating hairs are also extremely dangerous to humans, causing dermatitis and other severe reactions (Lamy 1990).

Increasing winter temperatures, such as those registered during the last two decades in Italy (Kumar et al. 2005; Morabito et al. 2007), seems to be favoring the PPM. In fact, since 1995 the PPM's range has been expanding constantly, in both latitude and altitude (Hódar et al. 2003; Hódar and Zamora 2004; Battisti et al. 2005; Robinet et al. 2013; Roques et al. 2015a). Moreover, in some areas, outbreaks are becoming more severe, as well as repeating over time (Hódar et al. 2012), as PPM larvae more readily feed with milder temperatures during the cold seasons (Hódar and Zamora 2004; Battisti et al. 2005; Hoch et al. 2009). This winter feeding reduces the mortality of overwintering larvae (Battisti et al. 2005; Buffo et al. 2007), while increasing both larval growth rate (Roques et al. 2015a) and females' fecundity (Markalas 1989; Pimentel et al. 2011). Battisti et al. (2005) have shown that in warmer winters larvae reach maturity more quickly and with higher reserves of energy. In fact, as PPM adults do not feed, all resources for reproduction come from larval feeding (Huchon and Démolin 1970), as do other lepidopterans (Gibbons et al. 1995; Oberhauser 1997; Williams 2011).

Summer temperatures are also important, as an increase may affect PPM development both positively and negatively. In fact, while, on the one hand, PPM females rarely emerge with average nighttime minimum temperatures lower than 14°C (Battisti et al. 2006; Bonsignore and Manti 2013), high summer temperatures may also negatively affect the PPM (Rouault et al. 2006). In fact, Huchon and Démolin (1970) have found that summer temperatures above 32°C, commonly occurring in the warmer areas of the PPM range, harm both PPM embryos and young larvae. However, the effects of high temperatures are still not clear (Roques et al. 2015b). As PPM's distribution range includes both seacoast and mountain pine stands, increasing summer temperatures will affect this species either positively or negatively depending on the area.

Although the PPM has various natural enemies (Battisti et al. 2000), egg parasitoids are one of the PPM's main natural control factors (Schmidt et al. 1999). Among the several egg parasitoid species, *Baryscapus servadeii* (Domenichini) (Hymenoptera, Chalcidoidea, Eulophidae) and *Ooencyrtus pityocampae* Mercet (Hymenoptera, Chalcidoidea, Encyrtidae) are the most recurring and have the highest parasitism rates (Bellin et al. 1990; Halperin 1990; Tiberi 1990; Tsankov et al. 1996; Schmidt et al. 1997; Mirchev et al. 1999). While the former is a specialist PPM parasitoid, the latter is a polyphagous parasitoid (Battisti 1989; Zovi et al. 2006; Cusumano et al. 2012; Mohammadpour et al. 2014). Other polyphagous species also parasitize PPM egg masses, such as *Anastatus bifasciatus* (Geoffroy) (Hymenotpera, Chalcidoidea, Eupelmidae) and *Trichogramma embryophagum* (Hartig) (Hymenoptera, Chalcidoidea, Trichogrammatidae), although they play a secondary role. All these species produce two generations in PPM eggs; the first generation emerges in summer shortly after PPM egg laying, while the second one emerges the following spring, after overwintering inside the host's eggs (Masutti 1964).

Climate change's effects on PPM egg parasitoids are little known, especially the effects on their distribution range or on their role in controlling PPM populations. Masutti (1964) stressed how temperature affects the development of both *O. pityocampae* and *A. bifasciatus*, as neither can endure temperatures above 30°C. *B. servadeii*, on the other hand, seems less susceptible to high temperatures (Masutti 1964; Tiberi 1990). This is confirmed by observations carried out in Israel and Morocco, where summer temperatures are quite high, with *O. pityocampae* mortality rates inside PPM eggs proving higher than *B. servadeii*'s (Kitt and Schmidt 1993; Schmidt et al. 1997). However, apart from the negative effects of excessive summer temperatures on some of the main PPM egg parasitoids, no other information is available on abiotic factors affecting parasitism.

The aim of our research was to study climatic factors affecting the PPM and its egg parasitoids. Specifically, the effects of temperature and rain fall on PPM fecundity and PPM egg parasitism were studied in a pine forest of Tuscany (Italy) over a 9‐year period. In particular, we tested the following: (1) whether PPM fecundity increases in mild winters; (2) whether PPM fecundity grows in summers with high minimum temperatures; (3) whether PPM egg abortion rates go up in hot summers; and (4) whether PPM egg parasitoids are negatively affected by both hot and rainy summers. The results of this study will help in predicting climate changes' effects on PPM outbreaks.

## Materials and Methods

Surveys were carried out from 2003 to 2011 in a PPM‐infested black pine stand, typical of submountainous pine forests in Tuscany, which had been planted in 1995 within the large Monte San Michele forest area (Greve in Chianti, Province of Florence, Italy) (43°33′N 11°22′E, 750–800 m a.s.l.). About 200 pines were set in rows spaced 4 m apart, perpendicular to contour lines, with pines every 3 m. The pine stand was facing south‐southwest, on a moderately steep arenaceous substrate. According to the local Forest Service, major PPM attacks had already been observed, starting from just 3 years after planting on. However, during the study period, Cayuela et al.'s infestation index (2014) was always low, with scant defoliation.

Each year, during January and February, each individual pine was carefully inspected for egg masses, which were then removed using a long‐reach pruner. All PPM egg masses found in the pine stand were collected and then taken to the laboratory. In addition, egg masses were located by searching in the vicinity of the first summer nest, normally built close to the egg mass. In the laboratory, the scaly cover of the egg masses was removed, then hatched and unhatched eggs were counted, identifying those parasitized by first‐generation parasitoids by their emergence holes. Immediately after, the egg masses were returned to outdoor conditions. Subsequently, each spring, the egg masses were taken back to the study area's pine stand and then fixed onto pine branches, at a maximum height of about 2 m, so that second‐generation parasitoids could contribute to the population dynamics of the local parasitoid community. Then, in summer, all egg masses were taken back to the laboratory, and unhatched eggs were dissected to record the cause of mortality: parasitized egg or aborted egg (dead embryo, dead PPM larva, or sterile egg). Parasitized eggs containing merely meconia were ascribed to egg parasitoid species according to Tanzen and Schmidt's methodology (1995). In this way, not only was total parasitism assessed but also that of each single parasitoid species. Parasitism rate was calculated considering both emerged parasitoids and dead ones inside the eggs.

Climatic data, kindly provided by ARSIA (Tuscany Region Agrometeorological Service) and SIR (Tuscany Region Hydrological Service), were recorded at the Lamole weather station (Greve in Chianti, 557 m a.s.l.), about 2.5 km from the study site.

### Statistical analysis

All the analyses were carried out using the R programming language (version 3.1.2; R Development Core Team 2014).

#### Egg mortality

Considering the zero‐inflated distribution of the data, different causes of egg mortality, expressed as percentages of PPM eggs over the whole study period, were compared using nonparametric tests. Parasitism rate and percentage of aborted eggs were compared using the Wilcoxon test. Differences within each group (parasitized and aborted eggs) were compared using Friedman's ANOVA followed by post hoc analysis (Wilcoxon).

#### Climate effects on PPM fecundity

Linear mixed effects models (LMEM) (Laird and Ware 1982) were developed, using the nlme R package (Pinheiro et al. 2014), to analyze the effect of climate‐independent variables (CIVs) on PPM fecundity (number of eggs per PPM egg mass), with year as a random variable and CIVs as fixed variables. Candidate models were selected by excluding those with correlated CIVs to avoid multicollinearity; the best model was then chosen based on Akaike's information criterion (AIC). CIV significance was determined by estimating the ML using the likelihood ratio test, following Zuur et al.'s protocol (2009).

After a careful review of the available literature, we found some climatic thresholds affecting PPM winter feeding and PPM flying activity; therefore, we evaluated whether they also affected PPM fecundity. As some climatic factors affect larvae's winter feeding (Battisti et al. 2005), and consequently adult females' fecundity (Markalas 1989), we included two CIVs recorded during the December–February trimester (the coldest months in our study area) prior to PPM egg laying. Firstly, we examined the number of days with a minimum temperature above 0°C, which is the lowest PPM feeding limit (Huchon and Démolin 1970) with temperatures inside the tent reaching at least 6°C the preceding day (Battisti et al. 2005). As tent temperatures are always 0.5–9°C higher than air temperatures (Breuer et al. 1989), we assume that, in our study site, tent temperatures were above 6°C when the preceding day's maximum air temperatures were close to 0°C Secondly, we included the average minimum daily temperatures, which had been suggested by Buffo et al. (2007) as a suitable index for PPM winter feeding. In addition, another CIV, the average minimum temperatures for the October–March period, indicated by Robinet et al. (2007) as the best predictor of the number of PPM feeding days, were also evaluated. Finally, the number of days with a temperature below 14°C for the July–August bimester was recorded: This coincided with the PPM flying period, as PPM females rarely fly below that temperature (Battisti et al. 2006), thus affecting PPM egg‐laying activity.

The model was validated by visual inspection of residuals (Zuur et al. 2009), which were also checked for homogeneity of variance, and absence of temporal autocorrelation. For this purpose, the GLS (generalized least squares) model with constant variance, and without temporal autocorrelation, was compared (through AIC) with: (1) one with a variance structure with a different spread per year (VarIdent structure; Zuur et al. 2009; Pinheiro et al. 2014); (2) one with temporal autocorrelation (AR‐1 correlation, corAR1; Zuur et al. 2009; Pinheiro et al. 2014); and (3) one with both (VarIdent and corAR1). The best model was finally tested using Bartlett's test.

#### Climate effects on egg mortality

All causes of egg mortality, having a zero‐inflated distribution, were treated as percentages of the number of eggs per egg mass. These, being nested within each study year, were then analyzed as dependent variables with GLMM binomial models (Zuur et al. 2009). The analyses were carried out using R package lme4 (Bates et al. 2014a,b), considering the year as a random factor, whereas PPM fecundity (a level 1 variable, i.e., a variable with different values for each egg mass; Zuur et al. 2009) and CIVs (level 2 variables, i.e., each explanatory variable has the same value for all egg masses within each year; Zuur et al. 2009) were included as fixed factors. The best model, with the smallest AIC value, was selected.

##### Egg abortion

The dependent variables considered were as follows: (1) percentages of eggs per egg mass containing dead PPM larvae or embryos and (2) percentages of sterile eggs per egg mass. As high temperatures negatively affects PPM egg survival (Huchon and Démolin 1970), the following CIVs recorded during the July–August bimester were considered: (1) the number of days with maximum temperatures above 30°C and (2) the average maximum daily temperature.

##### Egg parasitism

The dependent variables considered in this analysis were as follows: (1) percentage of total parasitized eggs per egg mass; (2) percentage of eggs (per egg mass) parasitized by *B. servadeii*, (3) *O. pityocampae*, (4) *A. bifasciatus,* and (5) *T. embryophagum*. As some PPM eggs parasitoids are negatively affected by high temperatures (Masutti 1964), we considered CIVs which may affect parasitoids during the hottest period (July–August for the study area), which is when PPM eggs are usually exposed to parasitism (Dulaurent et al. 2011). As for egg abortion, the CIVs were (1) number of days with maximum temperature above 30°C; (2) average maximum daily temperature; in addition, (3) cumulative rainfall in the same period was also considered, as rainfall may significantly limit parasitoid foraging behavior (Hilker and McNeil 2008).

## Results

### PPM fecundity and egg mortality

**Figure 2:**
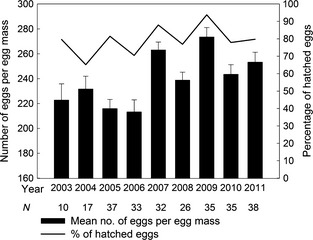
Average number of *Thaumetopoea pityocampa* eggs per egg mass and percentage of hatched eggs during the 2003–2011 samplings at the Monte San Michele pine stand (Greve in Chianti, Florence, Italy). *N* = total number of egg masses collected every year. Bars indicate standard errors.

**Figure 3:**
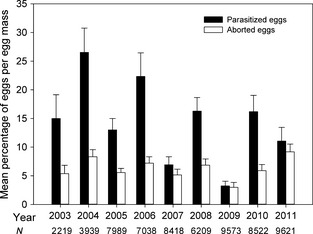
Mean percentages of *Thaumetopoea pityocampa*'s parasitized and aborted eggs at the Monte San Michele grove (Greve in Chianti, Florence, Italy). Bars indicate standard errors. *N* = total number of eggs per year.

PPM fecundity was quite variable during the study period, with parasitism representing the main cause of egg mortality. The mean number of eggs per egg mass, out of a total of 263 PPM egg masses, was 241.47 ± 2.99 SE, ranging from a minimum of 106 to a maximum of 365 in the whole study period. The lowest values were recorded during the first 4 years (2003–2006), while the highest were recorded from 2007 to 2011 (Fig. 2). The percentage of hatched eggs ranged from 65.15% in 2004 to 93.79% in 2009 (Fig. 2). The main cause of mortality for the entire period was parasitism (Wilcoxon, W = 39 998, *P* < 0.01). Indeed, aborted eggs amounted only to 6.23% (±0.38 SE), of which 2.31% (±0.21 SE) were sterile eggs and 3.93% (±0.02 SE) were dead PPM embryos or larvae (Table 1). In contrast, the mean parasitism rate per egg mass was 13.55% (±1.19 SE), ranging from 3.23% (±0.82 SE) to 26.53% (±4.23 SE) (Fig. 3).

**Table 1:** Mean percentages (±SE) of *Thaumetopoea pityocampa* parasitized and aborted eggs at Monte San Michele (Greve in Chianti, Florence, Italy) and climate‐independent variables (Lamole weather station – Greve in Chianti, Florence, Italy). DD > 0°C = number of days with minimum temperatures over 0°C for December–February; DD > 30°C = number of days with maximum temperatures over 30°C for July–August; RJ‐A = cumulative rainfall for July–August

Year	*Baryscapus servadeii*	*Ooencyrtus pityocampae*	*Anastatus bifasciatus*	*Trichogramma embryophagum*	Aborted eggs		Climate‐independent variables		
					Sterile eggs	Dead PPM larvae/embryos	DD > 0°C	DD > 30°C	RJ‐A
2003	4.56 ± 1.44	6.68 ± 2.15	3.14 ± 1.67	0.61 ± 0.56	1.29 ± 0.45	4.07 ± 0.09	63	32	36.5
2004	10.84 ± 1.90	12.72 ± 2.49	2.00 ± 0.47	0.97 ± 0.40	3.13 ± 0.75	5.19 ± 0.06	66	7	34.0
2005	6.02 ± 1.00	5.45 ± 0.87	1.10 ± 0.23	0.41 ± 0.11	2.24 ± 0.48	3.32 ± 0.03	52	8	130.0
2006	9.61 ± 1.99	10.93 ± 2.02	1.54 ± 0.51	0.25 ± 0.10	4.06 ± 0.80	3.13 ± 0.04	63	8	111.6
2007	2.42 ± 0.52	1.25 ± 0.39	2.95 ± 0.57	0.29 ± 0.08	1.91 ± 0.37	3.22 ± 0.04	88	13	100.2
2008	5.02 ± 0.93	4.60 ± 1.05	6.66 ± 1.01	0	1.56 ± 0.29	5.27 ± 0.07	78	21	60.0
2009	1.36 ± 0.35	0.72 ± 0.24	1.01 ± 0.29	0.13 ± 0.06	2.01 ± 0.74	0.97 ± 0.01	79	32	71.6
2010	7.39 ± 1.32	5.17 ± 0.98	3.24 ± 0.66	0.40 ± 0.11	1.16 ± 0.37	4.73 ± 0.06	75	3	71.4
2011	6.99 ± 1.73	2.55 ± 0.65	1.01 ± 0.22	0.50 ± 0.21	2.90 ± 0.69	6.28 ± 0.07	67	11	53.8
Total	5.89 ± 0.48	4.97 ± 0.44	2.33 ± 0.21	0.35 ± 0.05	2.31 ± 0.21	3.93 ± 0.02			

Friedman test χ^2^(3) = 298.983 *P* < 0.001. Post hoc pairwise comparisons (Wilcoxon W)			
	*O. pityocampae*	*A. bifasciatus*	*T. embryophagum*
*B. servadeii*	ns	43.500**	55.699**
*O. pityocampae*		40.243*	53.267**
*A. bifasciatus*			ns

Comparison of parasitism rates over the whole study period (level of significance: *P < 0.01; **P < 0.001).

Four egg parasitoid species were identified, all belonging to the Chalcidoidea superfamily: *A. bifasciatus*,* O. pityocampae*,* B. servadeii,* and *T. embryophagum*. Considering the whole study period, *B. servadeii* and *O. pityocampae* parasitism rates*,* although always below 13%, were significantly higher than those of *A. bifasciatus* or *T. embryophagum* (Table 1). Indeed, even on a year‐by‐year basis, *B. servadeii* and *O. pityocampae* were always the main parasitoid species, except for the 2007–2008 period, when *A. bifasciatus* was the most numerous species (Table 1).

### Climate effects on PPM fecundity

Mean annual maximum daily temperatures ranged from a minimum of 16.59°C (in 2010) to a maximum of 19.54°C (in 2008) during the 1999–2011 period. The lowest mean minimum daily temperature (8.75°C) was also recorded in 2010, while the highest (11.01°C) occurred in 2009. Thermal constancy was recorded over the 1999–2003 period; instead, a decrease was observed in 2004–2005, with a subsequent significant increase from 2006 to 2009. Regarding cumulative annual rainfall, the lowest values were recorded in 2003 and 2007 (622.50 and 633.60 mm, respectively), while 2010 registered the most rainfall (1382.80 mm).

**Figure 4:**
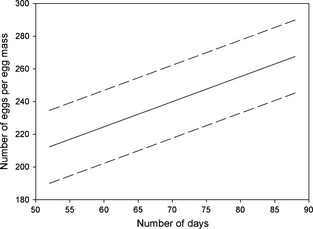
LMEM‐predicted number of *Thaumetopoea pityocampa* eggs per egg mass along the number of days (December–February) with minimum temperature above 0°C. Thick line in the middle represents the predicted values for the whole study period. Dotted lines (95% CI) were obtained by adding and subtracting 1.96 × SD (the random intercept) to/from the predictor function.

The number of days with temperatures above 0°C during the December–February period, which precedes PPM oviposition, (referred to as DD > 0°C) positively affected PPM fecundity (likelihood ratio test to estimate ML gave us L = 8.721, *P* = 0.0031). Indeed, based on AIC, the best model for PPM fecundity included DD > 0°C. DD > 0°C varied between 52 and 66 days during the December to February periods from 2002 to 2006, while it ranged from 63 to 88 days during the 2006–2011 December–February time frame (Table 1). PPM fecundity increased therefore in years with higher DD > 0°C; in fact, the model‐predicted mean number of PPM eggs per egg mass ranges from 212 in colder winters to 268 in milder ones (Fig. 4).

Visual inspection of residual plots did not reveal any obvious deviation from normality or homoscedasticity. In addition, regarding homogeneity of variance, Bartlett's tests were not significant either for the year variable (Bartlett's K‐squared = 10.9248, df = 8, *P*‐value = 0.206) or for the DD > 0°C variable (Bartlett's K‐squared = 9.3862, df = 7, *P*‐value = 0.2261).

### Climate effects on egg mortality

#### Egg abortion

No CIVs considered in the GLMM analysis of egg abortion were informative. Indeed, neither dead PPM larvae/embryos inside eggs nor sterile eggs resulted statistically related to the CIVs taken into consideration in our study area. On the other hand, PPM fecundity did affect egg abortion; in fact, both sterile eggs and dead larva/embryo percentages decreased with increasing number of eggs per egg mass (Table 2).

**Table 2:** GLMM best models selected according to AIC. DD > 30°C = number of days with maximum temperature over 30°C for July–August during the 2002–2011 period; RJ‐A = cumulative rainfall for July–August during the 2002–2011 period

Dependent variables	DD > 30°C		RJ‐A		PPM fecundity^a^	
	Estimate	*P*	Estimate	*P*	Estimate	*P*
Aborted eggs						
Sterile eggs					−0.1369	<0.001
Dead PPM larvae or embryos					−0.2037	<0.001
Rate of parasitism^b^						
*Baryscapus servadeii*	−0.4898	<0.001	−0.2422	0.0229	−0.1967	<0.001
*Ooencyrtus pityocampae*					−0.2550	<0.001
*Anastatus bifasciatus*					−0.1150	<0.001
*Trichogramma embryophagum*					−0.2586	<0.001
Total parasitism	−0.4160	0.0023	−0.2785	0.0042	−0.2326	<0.001

a Number of *Thaumetopoea pityocampa* eggs per egg mass.

b Number of *T. pityocampa* parasitized eggs per egg mass.

#### Egg parasitism

**Figure 5:**
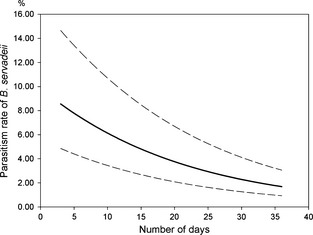
GLMM‐predicted probability of *Baryscapus servadeii* parasitism for the number of days (July–August) with maximum temperature above 30°C. Thick line in the middle represents the predicted values for the whole study period. Dotted lines (95% CI) were obtained by adding and subtracting 1.96 × SD (the random intercept) to/from the predictor function.

According to the GLMM results (Table 2), CIVs affected total egg parasitism. However, only two CIVs among those considered resulted significant, namely the number of days with temperatures exceeding 30°C during the July–August bimester (DD > 30°C), and the cumulative rainfall for the same time frame (RJ‐A). The total parasitism rate, being inversely related to these CIVs, decreased in years with more days over 30°C (DD > 30°C), as well as in those with higher rainfall (RJ‐A) (Table 2). The *B. servadeii* parasitism rate showed a similar pattern, although in this case the significance level was higher for DD > 30°C compared to RJ‐A (Table 2). Specifically, mean predicted *B. servadeii's* parasitism rate jumped from 1.69% in hotter summers to 8.55% in cooler summers (Fig. 5). On the contrary, no CIV effect on the other parasitoid species emerged from our analysis. However, all parasitism rates did decrease with increasing number of eggs per egg mass (Table 2).

The July–August period showed DD > 30°C values which are typical of mild‐summer regions; in fact, temperatures exceeded 30°C on more than 20 days over the July–August periods only in 2003, 2008, and 2009. Other than for these 3 years, such temperatures were never recorded for more than 13 days (Table 1). Furthermore, temperature highs were usually below 33°C, with only 4 days in the arc of 9 years reaching higher values, peaking at 37.1°C on one occasion. The RJ‐A factor had its minimum values in the 2003–2004 period (from 34 to 36.5 mm), while during the 2005–2007 period, its values increased dramatically (from 100.2 to 130 mm) (Table 1).

## Discussion

PPM infestation in our pine forest remained low throughout our whole study period. Specifically, the number of egg masses collected per year never exceeded one per five pines, which is the threshold for a low PPM density. In addition, the infestation index was low; in fact, pines were never completely defoliated, so food was always available for PPMs. Consequently, we assume that food availability and PPM density did not invalidate our results.

PPM fecundity in our study site was definitely affected by winter temperatures. The more PPM larvae feed during the winter, the larger females are going to be, and the more eggs they are going to lay (Markalas 1989; Gibbons et al. 1995; Oberhauser 1997; Pimentel et al. 2011; Williams 2011). Furthermore, our data reconfirm Battisti et al.'s results showing PPM larvae feed during winter when night lows are above 0°C and tent temperatures reach at least 6°C the preceding day (2005). As expected, in our study, DD > 0°C positively correlated to female fecundity, in agreement with Pimentel et al. (2011), who found a correlation between winter minimum temperatures, PPM female size, and their fecundity. In conclusion, milder winters facilitated larval feeding in the overwintering phase, so that the larvae reached maturity faster, and with substantial energy reserves to be spent on egg production.

Looking at the summer data, July–August minimum temperatures did not correlate with PPM fecundity, although temperature during mating and oviposition directly affects fecundity in other lepidopterans (Berger et al. 2008; Mahmoud 2013). However, average summer nighttime minimum temperatures below 14°C do affect PPM emergences negatively (Battisti et al. 2006; Bonsignore and Manti 2013). Consequently, we expected a negative upshot on PPM fecundity as well with temperatures below this threshold; however, no significant effect was actually observed. This may be due to the unavailability of data regarding nighttime average temperatures, as we only had the records of daily minimum temperatures. Furthermore, it may also be related to the fact that daily minimum temperatures only sporadically fell below 14°C in our study area, occurring on an average of 6 days during the July–August period every year. Thus, the temperatures recorded, whether due to the temperatures themselves or to the lack of nighttime logs, do not allow us to determine whether summer lows below 14°C correlate with PPM fecundity.

The percentage of hatching failure during the study period was quite low, with parasitism being the main mortality factor. Indeed, in our study site, unhatched eggs ranged from 6.21% to 34.85%, while in studies from other Mediterranean countries percentages were higher, ranging from 20 to 53% (Tiberi 1990; Tsankov et al. 1996; Schmidt et al. 1997, 1999; Pérez‐Contreras and Soler 2004; Castagneyrol et al. 2014). Among the causes of hatching failure, parasitism prevailed over aborted eggs; however, it was still lower than in other countries. Indeed, parasitism rate in our study ranged from 3.23 to 26.53%, while authors from other countries refer percentages from 4.5 to 38.9% (Bellin et al. 1990; Tsankov et al. 1996, 1998, 1999; Schmidt et al. 1997, 1999; Mirchev et al. 1999). Hence, being parasitism the main cause of egg mortality in our study, the quite low parasitism rates observed during the study period probably led to lower hatching failure compared with other studies' results.

PPM fecundity affected egg abortion, but no correlation was found between egg abortion and the climatic variables considered. As excessive summer temperatures are reported as leading to PPM egg mortality (Huchon and Démolin 1970; Rouault et al. 2006), we expected higher egg abortion in years with higher summer temperatures. However, maximum daily temperatures did not reach very high levels in our study area, rarely exceeding 32°C. Furthermore, some authors (Robinet et al. 2013) suggest that the upper temperature threshold for PPM larval development within eggs is actually much higher than 32°C; indeed, these authors did not find any correlation between heatwaves and PPM egg mortality. Regardless, such high summer temperatures were rarely recorded in our study area. Thus, similarly to Castagneyrol et al. (2014), we assume that abiotic factors do not play a major role in PPM egg survival in our study area. On the contrary, PPM fecundity affected egg abortion, as bigger egg masses had a lower egg abortion rate than smaller ones. Probably this is due to the females' health: Healthier females lay bigger egg masses, which also have a lower number of aborted eggs.

*Baryscapus servadeii* and *O. pityocampae* turned out to be, as expected, the main PPM egg parasitoids. This was true for most of the study years, except for 2007 and 2008, when *A. bifasciatus*, usually a minor PPM parasitoid (Masutti 1964; Battisti 1989; Tiberi 1990), had the highest parasitism rate. This sudden *A. bifasciatus* escalation may be explained by its adjustment to the eggs of *Leptoglossus occidentalis* Heidemann (Hemiptera, Coreidae). In fact, this exotic coreid, native to North America, was observed in our study area in significant population densities in 2007, although these then declined gradually over the following years (Niccoli et al. 2009). Consequently, after 2007–2008, *A. bifasciatus* went back to its role of secondary PPM parasitoid.

Both temperature and rainfall affected egg parasitism in our study area; in particular, parasitism was lower in years with warmer summers and in years with rainy summers. Specifically, the higher number of hot days (over 30°C) negatively affected total parasitism rate. This result is probably due to *B. servadeii*, which was the only parasitoid significantly affected by temperature. This is surprising, both because *B. servadeii* is otherwise known to be more temperature tolerant than the other parasitoid species (Masutti 1964; Tiberi 1990) and because temperatures above 30°C were infrequent in our study area. However, apart from its effects on survival, temperature may also affect parasitoids in different ways, such as by altering their synchrony with their hosts (Berggren et al. 2009; Thomson et al. 2010; Duan et al. 2014). Finally, *B. servadeii* parasitism was lower in years with rainy summers. As for the other parasitoids, rainfall likely caused a higher adult mortality, or it may have had a limiting effect on parasitoid foraging behavior (Hilker and McNeil 2008).

Our results confirm that climate warming affects insect outbreaks both directly and indirectly (Stireman et al. 2005; Berggren et al. 2009; Klapwijk et al. 2012; Ma et al. 2014). Milder winter temperatures in our study site directly favored the PPM by increasing its fecundity, in agreement with other studies (Klapwijk et al. 2012). Moreover, they indirectly favored the PPM, as their natural enemies, the parasitoids, did not have correspondingly higher rates. Furthermore, summer temperatures over 30°C negatively impacted *B. servadeii*, one of the main PPM parasitoids, yet did not hinder PPM egg development. This corroborates Stireman et al.'s findings (2005) that responses to climatic factors are species specific (Berggren et al. 2009; Ma et al. 2014); hence, climate changes affect parasitoids and their hosts differently, disrupting their enemy–herbivore dynamics.

Our results were obtained from a slightly infested pine forest, with high food availability for the PPM; however, we assume that climate warming will reduce *B. servadeii* parasitism also during large PPM outbreaks. Being *B. servadeii* a species‐specific parasitoid, it more closely depends on its host's density; consequently, it would be expected to increase its parasitism rate in times of PPM abundance (Klemola et al. 2010). However, parasitoid response to changes in host abundance is generally delayed (Taylor 1997), due to other mitigating factors. In the case of sudden PPM outbreaks, for example, PPM larval starvation might take place, due to their completely defoliating the host trees and a consequent lack of food, thereby depleting parasitoid host availability. In addition, severely and repeatedly defoliated pines become qualitatively unsuitable for PPMs, leading to higher larval mortality (Hódar et al. 2004) as well as waning fecundity and altered sex ratios (Awmack and Leather 2002). In this scenario, food availability and quality would be the main factors leading to PPM population collapse (Battisti et al. 2014), rather than an amplified parasitoid response.

In conclusion, we expect that climate changes, specifically ever‐milder winters, will favor PPM development by allowing it to spread into otherwise previously inhospitable environments, also triggering PPM outbreaks in areas where this pest's populations had previously been restrained by less favorable climate conditions and by higher parasitism pressure. However, as *B. servadeii* is the only parasitoid species significantly affected by high summer temperatures, the other parasitoids here studied may be able to counteract PPM expansion. As, unlike *B. servadeii*, these are not species‐specific parasitoids, they can live on other hosts besides the PPM. Thus, they may be able to escape the negative effects of the high temperatures that impede *B. servadeii* yet trigger increased PPM egg laying. Conversely, for exactly the same reason, they would survive periods of lower PPM population density. Therefore, they might thrive and ultimately be able to control the PPM in periods of higher temperatures.

## Conflict of Interest

None declared.
